# Chimeric antigen receptor macrophages therapy for glioblastoma: challenges and opportunities from preclinical evidence to clinical translation

**DOI:** 10.3389/fimmu.2026.1726329

**Published:** 2026-02-09

**Authors:** Qilong Zhai, Jingwen Cui, Zixiao Tan, Hongbin Wu, Yang Yu, Jiahang Sun

**Affiliations:** 1 The Second Affiliated Hospital of Harbin Medical University, Harbin, Heilongjiang, China; 2 Peking Union Medical College Hospital, Chinese Academy of Medical Sciences, Beijing, China

**Keywords:** cell therapy, chimeric antigen receptor macrophages, clinical translation, glioblastoma, immunotherapy, tumor microenvironment

## Abstract

Treatment failure in glioblastoma (GBM) is primarily attributed to the convergence of multiple barriers, including an immunosuppressive tumor microenvironment (TME), intratumoral heterogeneity, and the blood-brain barrier. Chimeric antigen receptor macrophages (CAR-M) therapy presents a promising new avenue for GBM treatment, leveraging its inherent tumor-homing capacity, TME reprogramming function, and potential to bridge innate and adaptive immunity. However, despite promising preclinical data, clinical efficacy in GBM remains unproven. This review critically analyzes the translational gap. We first outline the theoretical rationale and inherent advantages of CAR-M therapy in overcoming the core barriers of GBM. We then critically assess the limitations of current preclinical evidence and the uncertainties associated with its extrapolation to the clinical setting. We then focus on bottlenecks such as target selection strategies, engineering design, and TME-driven issues like phenotypic inactivation and antigen escape, discussing corresponding optimization approaches like armoring modifications, logic-gated designs, and convection-enhanced delivery. Finally, we propose a pragmatic clinical translation pathway prioritizing mechanistic validation. This pathway emphasizes integrating CAR-M therapy with combinatorial approaches and smart technologies in early-phase clinical trials, supported by biomarker analyzes, to address fundamental biological questions regarding the homing, survival, and function of these cells in patients. This review aims to provide a systematic and critical reference to guide the translation of CAR-M therapy from concept to clinical application, a path characterized by both opportunities and challenges.

## 1 Introduction

Glioblastoma (GBM), the most aggressive primary malignant brain tumor, continues to remain a formidable therapeutic challenge. Despite maximal safe resection followed by radiotherapy with concomitant adjuvant temozolomide (TMZ/RT-TMZ)-the standard of care for the past two decades—patient outcomes remain poor, with a median overall survival still under two years (1, 2). This therapeutic impasse primarily stems from the complex biological characteristics of GBM, including marked intratumoral heterogeneity, a highly immunosuppressive tumor microenvironment (TME), and the presence of the blood-brain barrier, which collectively constitute major obstacles to the development of effective therapies (2). Moreover, the GBM TME is not only profoundly immunosuppressive but also possesses unique properties distinct from other solid tumors. Within this *milieu*, tumor-associated macrophages (TAMs) and microglia represent the most abundant immune cell population, accounting for up to 50% of the total tumor cells (3). The composition and functional state of TAMs are closely correlated with molecular subtypes within Isocitrate dehydrogenase (IDH) -wildtype GBM (such as the Proneural, Mesenchymal, and Classical subtypes), representing a major aspect of TME heterogeneity (4). Furthermore, TAMs have a unique dual origin, comprising two distinct lineages: bone marrow-derived macrophages (BMDMs) and brain-resident microglia. Both lineages exhibit high plasticity within the TME (4). This complex cellular composition and the interactions among its components form the foundation of the GBM immune microenvironment, and the in-depth dissection of these interactive mechanisms remains a crucial direction for current research.

TAMs in GBM exhibit profound plasticity. While traditional views categorized them into a binary M1/M2 dichotomy, recent single-cell analyses reveal a complex, continuous spectrum of functional states that are predominantly immunosuppressive and tumor-supportive. This high abundance of TAMs, coupled with their inherent plasticity, provides the fundamental rationale for CAR-M therapy: rather than merely attempting to deplete these cells, CAR-M technology aims to precisely reprogram them from pro-tumorigenic accomplices into stable anti-tumor effectors.

Based on the central role of TAMs in the GBM TME, reshaping the TME through immunotherapy has become a critical strategy. However, despite the success of immune checkpoint inhibitors (ICBs) and chimeric antigen receptor T-cell (CAR-T) therapies in various cancers, their clinical benefit in GBM remains extremely limited (5, 6). These clinical trials clearly revealed that overcoming the immunosuppressive microenvironment of GBM is a prerequisite for the success of any novel immunotherapy.

These therapeutic challenges have spurred the exploration of alternative cell-based therapies, among which chimeric antigen receptor macrophages (CAR-M) therapy has garnered significant attention. Unlike primarily lymphocyte-based approaches, the core value of the CAR-M platform lies in the inherent biological properties of macrophages: their capacity for tissue infiltration, functional plasticity, and their role as a bridge between innate and adaptive immunity. This positions CAR-M to theoretically address the multiple barriers of GBM in a coordinated manner, with a mechanism of action that extends beyond simple direct cytotoxicity. As innate tissue infiltrators and innate immune effector cells, macrophages possess unique advantages in solid tumor treatment (7, 8). First, preclinical studies indicate that they can be recruited by tumor-secreted factors (e.g., CCL2, CSF1) (9) and infiltrate into the tumor core and hypoxic regions, suggesting a potential to overcome the migration and infiltration hurdles that often limit CAR-T cells in solid tumors (10, 11). This inherent chemotactic ability theoretically positions them to traverse the blood-brain barrier and facilitate intratumoral accumulation. Second, macrophages possess potent innate immune functions, including phagocytosis (12, 13), direct cytotoxic activity, and antigen presentation. Crucially, their intrinsic plasticity allows for the theoretical possibility of using CAR signaling to sustain them into a potent M1-like anti-tumor phenotype (14–16). This engineered polarization represents a strategy designed to resist the suppressive TME, with the goal of enabling the cells to sustain active tumor attack and remodel the immune microenvironment (11). This provides a rational basis for directly reprogramming the abundant TAMs in GBM to alleviate immunosuppression. Collectively, these attributes establish CAR-M as a promising candidate strategy for treating GBM and other solid tumors. Their unique macrophage characteristics, particularly phagocytosis, confer a distinctive value compared to CAR-T and chimeric antigen receptor natural killer cell (CAR-NK) therapies. Specifically, their phagocytic capabilities offer a mechanism for direct clearance of heterogeneous tumor cells, while their antigen-presenting function is hypothesized to prime adaptive immunity against non-targeted antigens. If validated, this process could facilitate epitope spreading, thereby addressing the challenge of antigen escape. Consequently, CAR-M holds significant potential for driving robust anti-tumor immunity in solid tumors.

In summary, the therapeutic impasse in GBM stems from its unique triple barriers, while CAR-M therapy, leveraging its inherent biological properties, emerges as a conceptually promising platform to address these challenges. However, translating its preclinical theoretical promise into clinical reality faces a series of formidable obstacles, spanning cell design, target selection, *in vivo* delivery, and safety control. Currently, no clinical efficacy data for CAR-M in glioma patients have been reported globally, rendering the future development of this field both promising and fraught with uncertainty. This data vacuum necessitates a critical appraisal of the existing preclinical evidence and the charting of a pragmatic translational path.

Diverging from narratives that primarily emphasize its advantages, this review aims to construct a systematic analytical framework spanning from preclinical evidence to clinical translation. We will first scrutinize the fundamental challenges confronting CAR-M therapy in GBM, including the inherent limitations of preclinical models, TME-driven functional inactivation of cells, and dynamic target evolution. Building upon this critical assessment, the article will explore potential breakthrough opportunities, such as next-generation intelligent engineering strategies, synergistic effects of combination therapies, and the unique safety window offered by local delivery. Through this critical synthesis and forward-looking perspective, this review seeks to bridge the gap between laboratory discoveries and clinical application, providing a reference that integrates scientific insight with translational pragmatism, with the ultimate goal of steering CAR-M therapy toward a more robust and rational clinical validation phase.

## 2 Methods

To comprehensively review the research progress of chimeric antigen receptor macrophages (CAR-M) in glioblastoma (GBM), we performed a systematic search of electronic databases including PubMed, Web of Science, and Embase from their inception until September 2025. The search strategy combined the following core keyword combination: (“CAR-M” OR “chimeric antigen receptor macrophages”) AND (“glioma” OR “glioblastoma”) AND (“immunotherapy” OR “tumor microenvironment”). The literature selection prioritized original research focusing on CAR-M engineering, mechanistic studies in GBM models, and relevant clinical data from other solid tumors that inform intracranial applications. We also included key reviews to contextualize the field. Studies were critically evaluated based on the relevance of their experimental models and the rigor of their reporting, particularly concerning the validity of recapitulating the GBM microenvironment. Irrelevant studies, conference abstracts lacking sufficient data, and non-peer-reviewed publications were excluded.

## 3 Rationale and inherent advantages of CAR-M for confronting GBM

The therapeutic challenge in GBM stems from its multi-layered defense system, which includes the physical blood-brain barrier, the cellular immunosuppressive TME, and the genetic intratumoral heterogeneity. CAR-M therapy demonstrates significant potential because its biological characteristics are theoretically well-suited to overcome these barriers and can be precisely enhanced through engineering strategies. However, it should be clearly stated that many of the advantages discussed below are currently largely inferred from preclinical studies, and their efficacy in the complex human environment awaits clinical validation. In comparison to lymphocyte-based therapies like CAR-T and CAR-NK, the CAR-M platform offers several potential advantages in tumor infiltration, phenotypic plasticity, and systemic immune activation, as summarized in **Table 1**. Nevertheless, its inherent cellular characteristics, such as limited expansion capacity and potential pro-fibrotic risk, also present unique translational challenges.

**Table 1:** Comparison of CAR-M, CAR-T, and CAR-NK cell therapies for GBM.

Feature dimension	CAR-M	CAR-T	CAR-NK
Tumor homing and infiltration	Can actively cross the BBB *via* native chemotactic axes such as CCR2/CCL2 (23, 24).	Poor migration and infiltration in solid tumors (25).	Homing capacity is less well-defined (26).
Primary killing mechanism	Phagocytosis; direct cytotoxicity (27).	Cell cytotoxicity such as perforin/granzyme (28).	Cell cytotoxicity (29, 30).
Phenotypic plasticity and stability	High plasticity; potential for sustained M1-like polarization *via* engineered signaling (31).	Prone to functional exhaustion within the TME (32).	Phenotypic stability requires further investigation (33, 34).
Antigen presentation and immune activation	Professional antigen-presenting cells; potential for cross-presentation and “bystander effect (35, 36).”	Weak antigen presentation capacity (37).	Antigen presentation capability is generally weaker than professional APCs (38, 39).
Primary potential risks	Potential pro-fibrotic risk; intracranial hyperinflammation (35, 40).	Cytokine release syndrome (CRS); immune effector cell-associated neurotoxicity syndrome (ICANS) (41, 42).	Toxicity is generally milder (38).

This table summarizes the potential advantages and challenges of CAR-M therapy relative to other CAR-engineered cell therapies, based on their inherent biological properties as discussed in the manuscript.

### 3.1 Innate homing capacity to overcome delivery barriers

The blood-brain barrier (BBB) constitutes the primary physical obstacle for systemically administered cellular therapeutics. Unlike traditional lymphocytes, macrophage precursors—monocytes—naturally express high levels of chemokine receptors such as CCR2 and CSF1R. These receptors correspond to ligands (e.g., CCL2 and CSF-1) that are abundantly secreted by GBM cells and within the TME (10, 11). Mechanistically, this inherent chemotactic axis, coupled with the secretion of matrix metalloproteinases (MMPs) that degrade the extracellular matrix (17), provides the biological basis for CAR-M to sense chemical gradients. Preclinical models suggest this allows them to theoretically traverse the BBB or accumulate at sites of disruption, facilitating “homing” from the circulation into the tumor parenchyma (18–20). However, extrapolating these findings to humans requires caution. Unlike the relatively uniform BBB disruption seen in syngeneic murine models, human GBM vasculature exhibits significant spatial heterogeneity, where regions of intact BBB may still impede macrophage infiltration (21, 22). Consequently, while this active infiltration capacity represents a key theoretical advantage, its efficiency in achieving therapeutic cell density throughout the entire tumor volume in patients remains to be validated, distinguishing it from the absolute barriers that have historically limited the efficacy of CAR-T therapies in solid tumors.

### 3.2 Phenotype maintenance against immunosuppressive reprogramming

Following successful cellular infiltration, the next critical challenge for CAR-M therapy is maintaining durable and stable anti-tumor function. The GBM TME constitutes a profound immunosuppressive niche that can exhaust infiltrating immune cells or drive them toward pro-tumor phenotypes. The high plasticity of macrophages presents a double-edged sword, and CAR-M technology aims to harness this plasticity. By incorporating specific intracellular signaling domains (e.g., ITAM motifs derived from FcRγ or DAP12), CAR-M are designed to initiate the SYK-PI3K/MAPK signaling pathway upon antigen recognition. This pathway preferentially drives phagocytosis and pro-inflammatory cytokine expression, thereby polarizing the cells towards a stable M1-like phenotype (43). The strategic selection of this signaling circuitry is designed to counteract inhibitory factors like TGF-β (44), which are abundant in the GBM TME, with the aim of sustaining anti-tumor functionality. This engineered phenotype maintenance may allow CAR-M to withstand the inhibitory conditioning of the GBM TME, maintaining potent cytotoxic activity within this hostile microenvironment. Furthermore, activated CAR-M secrete high levels of pro-inflammatory cytokines such as IFN-γ and TNF-α, which not only directly kill tumor cells but have also been shown in preclinical studies to repolarize surrounding pro-tumor M2-like TAMs into anti-tumor M1-like phenotypes *via* paracrine effects. Nevertheless, this interplay is dynamic and bidirectional. Emerging evidence indicates that chronic exposure to high concentrations of TGF-β and IL-10 can eventually dampen CAR signaling, potentially forcing even activated cells into a dysfunctional state (45). Thus, while broad TME remodeling is a potential benefit, maintaining phenotype durability against such overwhelmed suppression remains a formidable translational hurdle.

While sustained inflammatory polarization is a core objective, it remains one of the most formidable translational hurdles. Current evidence supporting phenotype stability largely originates from short-term *in vitro* systems or immunodeficient mouse models, which cannot fully recapitulate the chronic, multi-factorial suppressive pressure of the human GBM TME. Consequently, whether the short-term pro-inflammatory phenotype observed in preclinical studies can persist for weeks to months in patients without succumbing to functional exhaustion or epigenetic silencing remains a pivotal uncertainty. Furthermore, within the sensitive intracranial environment, balancing this sustained potency with safety is crucial; prolonged persistence of a highly inflammatory M1 phenotype carries the risk of chronic neuroinflammation, necessitating future designs that may require programmable safety switches to ensure the timely resolution of immune responses after tumor clearance (46, 47).

### 3.3 Countering tumor heterogeneity: from direct killing to systemic immune activation

The extreme intratumoral heterogeneity of GBM means any therapy targeting a single antigen inevitably faces the challenge of antigen escape. The core potential of CAR-M therapy lies in its ability to transcend the conventional “one-to-one” killing paradigm. First, specific phagocytosis enables direct clearance of antigen-positive tumor cells, representing the most direct killing mechanism (48). Second, another fundamental theoretical potential stems from CAR-M’s role as professional antigen-presenting cells (APCs). After phagocytosing tumor cells, CAR-M is hypothesized to process and cross-present tumor antigens, potentially activating tumor-specific T cell responses. This mechanism aims to generate a “bystander effect”—immunologically termed epitope spreading—that eliminates tumor cell clones lacking the target antigen (16, 49). This process provides a theoretical framework to address GBM heterogeneity (50) by recruiting the adaptive immune system to target the broader mutational landscape of the tumor. If validated in humans, this process would function as a personalized “*in situ* vaccination”. It is important to acknowledge, however, that this mechanism relies heavily on a responsive host immune system. Given that GBM patients frequently suffer from systemic T-cell lymphopenia and exhaustion—often exacerbated by standard-of-care radiochemotherapy—the capacity of CAR-M to successfully prime an unresponsive adaptive immune system represents a critical variable determining clinical efficacy (51, 52).

## 4 Engineering design of CAR-M for GBM

The design of CAR-M for GBM should move beyond generic approaches and instead be tailored to the pathophysiological characteristics of GBM. From core CAR signaling design to cell sources and manufacturing processes, each choice significantly impacts efficacy and safety within the complex GBM environment while also correlating closely with potential clinical risks.

### 4.1 CAR structural design: tailoring for phagocytosis and killing in GBM

Although CAR-M share the basic architecture of CAR-T cells, their structural design requires specific adaptations for macrophage biology. First, the affinity of the extracellular antigen-binding domain should be precisely calibrated to prevent on-target/off-tumor toxicity (53, 54). Second, unlike T cells, macrophages require a sufficiently long hinge region to provide the mechanical flexibility needed for phagocytosing large tumor cells (16, 55–57). However, the most fundamental distinction lies in the intracellular signaling domains. Macrophages differ essentially from T cells in their effector functions. Unlike T cells, simply adopting a CD3ζ-dominated signaling architecture may fail to maximize the therapeutic potential of CAR-M. Preclinical research indicates that targeted activation of the intrinsic intracellular signaling pathways of macrophages may be key to optimizing their anti-tumor efficacy. For instance, studies demonstrate that CAR designs preferentially activating innate macrophage signaling pathways—such as those utilizing transduction domains based on FcRγ or DAP12—can, in murine models, more effectively initiate phagocytosis programs and drive pro-inflammatory polarization upon antigen recognition (27, 58, 59). This mechanism is critically linked to the potential for achieving the core pro-inflammatory phenotype maintenance (35, 55).

This structural optimization is particularly relevant for targeting resistant subpopulations. Notably, glioma stem cells (GSCs), which are responsible for tumor recurrence and therapy resistance, often form cellular clusters (60, 61) or exhibit resistance to conventional phagocytosis. Consequently, exploring how to optimize the CAR structure to enhance the clearance of challenging targets like GSCs represents a crucial future direction. Future research should investigate whether, beyond the classical FcRγ signaling, other intracellular signaling pathways associated with large particle phagocytosis—specifically those derived from the engulfment receptors Megf10 or MerTK—can be integrated to theoretically augment the phagocytic capacity of CAR-M. However, such concepts remain entirely speculative at this stage. Their feasibility, the significant metabolic burden they might impose, and the potential risks of off-target effects on normal tissues are all major unresolved questions requiring urgent exploration.

### 4.2 Cell source selection: addressing the clinical reality of GBM patients

The clinical translation of CAR-M therapy critically depends on stable, scalable sources of macrophages. Current primary cell sources for CAR-M construction include peripheral blood mononuclear cells (PBMCs), hematopoietic stem and progenitor cells (HSPCs), and induced pluripotent stem cells (iPSCs).

PBMCs, due to their relative ease of isolation from peripheral blood, were the earliest cell type used for CAR-M generation (10). However, for recurrent GBM patients who have undergone multiple surgeries, temozolomide chemotherapy, and radiotherapy, their autologous PBMCs may suffer from insufficient quantity, functional impairment, or cellular senescence, thereby compromising the quality and efficacy of the final cell product. This clinical reality significantly limits the application prospects of autologous PBMC-derived CAR-M. To overcome this limitation, utilizing HSPCs or iPSCs with greater differentiation potential as novel cell sources for CAR-M suggests a paradigm shift from patient-specific autologous treatments towards pre-manufactured, cryopreserved, off-the-shelf allogeneic approach (62, 63). Among these, iPSCs have garnered significant attention as a source for CAR-M. Directed differentiation from a single clonal iPSCs line can generate an unlimited number of genetically identical macrophages (55, 64). This approach thus holds the potential to overcome the critical limitations of source scarcity and compromised cell quality in heavily pretreated patients, while also enabling unprecedented product standardization and off-the-shelf availability (27, 65, 66). Despite these manufacturing advantages, whether iPSC-derived macrophages are functionally equivalent to their physiological counterparts remains a subject of ongoing investigation. Comparative transcriptomic analyzes suggest that iPSC-derived lineages may exhibit distinct adhesion and chemotactic profiles compared to primary blood-derived macrophages, potentially impacting their *in vivo* migration efficiency (64, 67). Unlike the relatively straightforward processing of autologous cells, the production of iPSC-derived CAR-M necessitates a rigorous, multi-stage workflow compliant with Good Manufacturing Practice (GMP). This process should start from a clinically validated Master Cell Bank (MCB) to ensure consistency. The manufacturing pipeline typically involves feeder-free reprogramming, gene editing, and a directed differentiation cascade mimicking embryonic myelopoiesis.

To satisfy regulatory scrutiny, particularly referencing the precedent established by the first-in-class CT-0508 IND application, the release testing criteria should be exceptionally stringent. While CT-0508 validated the safety profile and potency metrics (phagocytosis and cytokine release) of CAR-macrophages [3], iPSC sources introduce unique risks that demand a zero-tolerance policy for residual undifferentiated cells. Consequently, release testing should integrate highly sensitive molecular assays (e.g., qRT-PCR or flow cytometry for Oct4, Nanog, and TRA-1-60) alongside standard sterility and endotoxin tests to rule out teratoma formation risks [4]. Furthermore, regarding functional potency—a critical component of the IND package—developers should adopt the validated metrics from the CT-0508 experience, which prioritize quantitative phagocytosis assays and cytokine secretion profiles (e.g., IFN-γ/IL-12 vs. IL-10 ratios) over simple expansion rates.

A critical limitation currently overlooked is genomic stability during the scale-up phase. The extended culture period required for iPSC differentiation increases the susceptibility to genetic drift and copy number variations (CNVs). Therefore, future clinical protocols should mandate high-resolution karyotyping and whole-genome sequencing not just on the MCB, but as part of the final product release criteria. Only by establishing such a comprehensive quality-by-design framework can iPSC-CAR-M truly transition from a promising concept to a standardized off-the-shelf biologic for GBM patients.

### 4.3 Manufacturing process: balancing efficacy with safety

Following the identification of a cell source, the next critical step is to convert these cells into therapeutic products through effective genetic transduction and manufacturing processes. In CAR-M production, the efficiency of genetic transduction is a pivotal factor determining functional success. Due to their intrinsic phagocytic and viral degradation capabilities, macrophages exhibit considerable resistance to transduction with commonly used viral vectors, such as lentiviruses, generally resulting in low transduction efficiency (66).

Furthermore, these cells present similar barriers to non-viral vectors, including mRNA, which further complicates their genetic modification (68). Lentiviral vectors can stably integrate CAR genes into the host genome, enabling long-term persistent CAR expression (16, 69). However, the risk of insertional mutagenesis associated with genomic integration, albeit relatively low in probability, remains a serious safety concern in clinical translation, particularly in sensitive tissues like the central nervous system.

In contrast to integrating viral vectors, mRNA electroporation introduces *in vitro* transcribed mRNA into cells *via* electroporation, offering superior safety and controllability of expression (68). This technique mediates transient CAR expression, typically lasting 5–7 days before natural degradation of the mRNA. This inherent characteristic provides a built-in biosafety switch that can automatically terminate CAR function in the event of severe adverse effects (70). Given these safety advantages, mRNA electroporation is more readily acceptable to regulatory agencies and has emerged as the preferred strategy for advancing first-in-human clinical trials. While the clinical safety of CAR-M therapy has been preliminarily validated by the adenoviral-based CT-0508 trial, the transient expression profile of mRNA offers a distinct layer of controllability (70). However, the transient nature of CAR expression imposes a fundamental limitation on the durability of efficacy, potentially necessitating complex multi-dosing regimens to compensate, thereby introducing new challenges for treatment implementation and accessibility.

Given that CAR-M therapy for GBM will likely utilize intracranial administration strategies such as convection-enhanced delivery (71, 72), ensuring safety within the confined intracranial space is paramount. In this context, CAR-M products utilizing transient mRNA transduction—characterized by their lack of genomic integration risk and self-limiting expression profile—provide a critical safety foundation for first-in-human clinical trials. This transient expression strategy is particularly well-suited for exploration in the sensitive environment of the central nervous system, maximally controlling potential risks of excessive inflammatory responses. However, a critical limitation should be acknowledged: following the decay of CAR mRNA, the macrophages are not eliminated but remain within the tumor. Without the sustained activation from CAR signaling, these cells may be susceptible to the suppressive TME, raising the concern of potentially reverting to a pro-tumor M2-like phenotype (73, 74). This risk of phenotypic reversion constitutes a substantial drawback, implying that while mRNA transfection offers a safety buffer for initial trials, it may compromise long-term efficacy or even inadvertently support tumor progression.

In summary, developing CAR-M to target GBM necessitates a fundamental shift in design logic—from universal technological application to disease-specific solutions. This implies that CAR signaling domains should prioritize phagocytosis activation over mere cytotoxicity; cell sources should address the clinical reality of functionally compromised cells in GBM patients by exploring off-the-shelf sources like iPSCs; and manufacturing processes should prioritize safety for intracranial therapy by balancing persistence with controllability. Only through the integrated and synergistic optimization of structure, source, and manufacturing can a solid foundation be established for subsequent clinical translation.

## 5 Target selection strategy for CAR-M in GBM

Target selection is the determinant of therapeutic specificity and safety in CAR-M therapy (57). An ideal target should balance high specificity with sufficient expression levels to overcome the highly heterogeneous TME. Beyond expression profiles, the target strategy requires a critical synthesis of mechanistic synergy, molecular stratification, and clinical druggability to maximize the therapeutic window (35, 55, 75).

### 5.1 Logical framework for target evaluation

We established a multidimensional evaluation framework to prioritize candidates. First, molecular stratification is essential; target expression should ideally correlate with prognostic markers such as IDH mutation status and MGMT promoter methylation to identify responsive patient cohorts (76–78). Second, functional synergy is critical. Unlike lymphocyte-based therapies, CAR-M targets should facilitate macrophage-specific functions—phagocytosis and TME remodeling—rather than merely serving as binding anchors (10, 79). Finally, translational feasibility should be assessed by referencing safety data from existing ADC or CAR-T trials to mitigate clinical risks (80–83).

### 5.2 In-depth analysis and strategic positioning of core candidate targets

We conducted a comparative analysis of leading candidates (summarized in **Table 2**) to define their strategic roles.

**Table 2:** Evaluation of selected candidate targets for CAR-M therapy in GBM.

Target	Expression and molecular stratification	Strategic rationale	Key challenges
B7-H3	High expression and low heterogeneity; expression is enriched in the Mesenchymal subtype (95, 103).	Dual mechanism combining phagocytosis with disruption of a key immunosuppressive pathway (104, 105).	On-target/off-tumor toxicity is a concern due to its physiological expression in some normal tissues (95, 106).
HER2	Overexpressed in approximately 80% of glioblastoma cases (1).	Offers a de-risked translational pathway supported by clinical validation such as the CT-0508 trial (107).	Requires careful definition of the therapeutic window (103).
EGFRvIII	Exhibits exceptionally high tumor specificity but also high intratumoral heterogeneity; enriched in the Classical subtype (84, 85, 103).	Serves as an ideal catalyst to initiate the CAR-M platform and an *in situ* vaccination effect (91).	Prone to dynamic downregulation leading to antigen escape (103, 108).
IL13Rα2	Overexpressed in a high percentage of glioblastoma cases with levels positively correlating with tumor grade (103).	Clinical precedent from CAR-T therapy supports its feasibility and informs local delivery strategies (6, 109).	Its complex biological role and weaker association with molecular subtypes pose challenges (110–113).
GSCs	Expressed on glioma stem cells; characterized by low abundance and high heterogeneity (114, 115).	Represents a foundational strategy to target tumor-initiating cells at the root of recurrence (116, 117).	Target expression is highly heterogeneous and dynamic; potential toxicity to normal stem cells requires evaluation (95, 118, 119).

This table provides a comparative overview of several target antigens based on their expression, strategic rationale, and associated challenges as described in the text.

#### 5.2.1 B7-H3 and EGFRvIII: balancing target stability and specificity

A comparative analysis of B7-H3 and EGFRvIII reveals a critical trade-off between tumor specificity and antigen stability. EGFRvIII is characterized by exceptional specificity and is typically enriched in the Classical GBM subtype (84–86). However, its utility as a standalone target is limited by marked intratumoral heterogeneity and a propensity for rapid downregulation following treatment (87–89). Therefore, rather than serving as a driver for long-term clonal clearance, EGFRvIII is strategically positioned as a catalyst to safely activate the CAR-M platform and prime systemic immunity *via in situ* vaccination during the initial treatment phase (90, 91).

In contrast, B7-H3 demonstrates superior stability and broader therapeutic potential. Unlike EGFRvIII, B7-H3 maintains high expression in recurrent GBM and is resilient to conventional radiochemotherapy (92, 93). Crucially, B7-H3 is enriched in the Mesenchymal GBM subtype (94, 95), offering a targeted approach for this aggressive population. Mechanistically, targeting B7-H3 provides a dual advantage uniquely accessible to CAR-M: it not only mediates direct phagocytosis but also disrupts the non-canonical immune checkpoint function of B7-H3, which drives macrophages toward an immunosuppressive M2 phenotype *via* the STAT3 pathway (35, 96–98). Furthermore, because B7-H3 is broadly expressed on both tumor cells and the tumor vascular stroma (99, 100), B7-H3-targeted CAR-M can sustain anti-tumor pressure by disrupting the tumor-supportive niche. However, this broad expression profile also necessitates careful safety profiling. Since macrophages are long-lived tissue-resident cells, low-level basal expression of B7-H3 in normal peripheral tissues (e.g., liver, adrenal gland) could pose a risk of on-target/off-tumor toxicity that differs from the transient kinetics of T cells (101, 102).

#### 5.2.2 HER2 and IL13Rα2: clinical validation and delivery strategies

From the perspective of clinical translation, HER2 and IL13Rα2 represent the most de-risked candidates, though they offer different strategic advantages regarding safety and delivery.

HER2 provides a robust, risk-controlled pathway supported by a well-established clinical translation foundation. It is overexpressed in approximately 80% of GBM cases (1) and currently represents the most clinically validated target within the CAR-M platform. Specifically, the CT-0508 clinical trial has provided preliminary evidence of the safety and feasibility of HER2-targeted CAR-M in solid tumors (107). Although its expression is not significantly enriched in any specific molecular subtype (120), its broad expression profile makes it a reliable universal target for GBM, validated by existing clinical evidence (107).

IL13Rα2, conversely, highlights the critical importance of delivery strategy. Its expression levels positively correlate with tumor grade in diffuse gliomas, making it highly relevant for high-grade cases (92, 121). The strongest argument for IL13Rα2 lies in the solid clinical precedent set by CAR-T therapies (NCT02208362). In this landmark study, IL13Rα2-targeted therapy administered *via* intratumoral delivery demonstrated encouraging signals of efficacy, including documented cases of tumor regression (109). Importantly, this offers a critical insight for CAR-M: local intracranial delivery represents a fundamental strategy to circumvent tumor infiltration barriers and initiate potent anti-tumor immunity (122). Although the biological role of IL13Rα2 is complex and its association with specific molecular subtypes is less defined than for other targets (123), its prevalent overexpression pattern combined with existing clinical success establishes it as a promising broad-spectrum candidate tailored for local delivery strategies.

### 5.3 Addressing the root of recurrence: targeting glioma stem cells

Targeting GSCs represents a fundamental strategy to curb tumor recurrence at its root (116, 117). Successful targeting and clearance of GSCs could theoretically undermine the long-term regenerative capacity of tumors. Mechanistically, the potent physical phagocytic capacity of CAR-M is highly aligned with the need to eliminate these core tumorigenic populations. Notably, the GSC phenotype is dynamically influenced by GBM molecular subtypes; for instance, the Mesenchymal GBM subtype is often enriched with GSC subpopulations exhibiting aggressive, therapy-resistant characteristics (124, 125). Thus, future strategies may need to be integrated with patient molecular subtyping to enhance therapeutic precision.

However, this strategy faces considerable challenges beyond simple identification. First, some markers are expressed at low levels in normal tissues (e.g., hematopoietic stem cells), necessitating careful evaluation of on-target/off-tumor toxicity (126, 127). Second, and most critically, GSCs possess remarkable phenotypic plasticity; they are not a static population but can dynamically transition between states such as “quiescent” and “proliferative” or “mesenchymal” and “pro-neural.” This leads to highly heterogeneous and fluctuating expression of surface markers (128–131). Consequently, therapies targeting a single GSC marker are highly susceptible to failure due to state transition or clonal selection. Currently, a critical knowledge gap lies in the lack of direct quantification of CAR-M phagocytosis efficiency *in vivo*. Developing high-resolution live imaging techniques to real-time track CAR-M/GSC interactions will be an essential tool for validating the efficacy of this strategy.

In summary, the target strategy for CAR-M in GBM constitutes a complex systems engineering task based on multidimensional evaluation, where no single one-size-fits-all solution exists. Currently, the landscape offers distinct therapeutic tools: HER2 and IL13Rα2 provide the most substantiated translational pathways based on clinical safety data; B7-H3 demonstrates considerable potential due to its mechanistic synergy in remodeling the TME; while EGFRvIII, despite its instability, serves as an ideal model for validating the bystander effect.

Future target exploration should transition from a static target-shooting mindset to a dynamic systems management approach. This implies that beyond optimizing single targets, greater emphasis should be placed on developing next-generation strategies capable of proactively addressing tumor heterogeneity and evolution (118, 119). Examples include constructing “OR”-logic gated CAR-M that simultaneously recognize dual tumor-associated antigens thereby establishing a functionally redundant recognition system to counter antigen escape (132–134). Although the precision and safety of such complex designs in humans remain substantial challenges, this combinatorial evolution undoubtedly represents the essential pathway toward achieving comprehensive and fundamental suppression of GBM.

## 6 Core challenges in clinical translation and mitigation strategies

The clinical translation of CAR-M therapy in GBM fundamentally depends on whether the engineered cells can maintain durable anti-tumor function within the highly immunosuppressive TME. Therefore, overcoming TME-induced functional suppression is widely considered the primary barrier that should be breached to address subsequent obstacles. This section systematically analyzes the key translational bottlenecks amplified by the TME, critically reviewing the synergistic rationale and inherent limitations of corresponding optimization strategies. **Table 3** provides a structured overview of these major translational bottlenecks, alongside the current leading optimization strategies and a critical appraisal of their associated limitations.

**Table 3:** Key challenges and discussed optimization strategies for CAR-M clinical translation in GBM.

Core challenge	Optimization strategy	Potential limitations and risks
TME-induced phenotypic deactivation and functional exhaustion	Armoring modifications including SOCS1 knockout, IRF5 or PPARγ overexpression, and constitutive expression of IL-12, IFN-γ or CD40L (35, 155–158).	Risk of uncontrollable neuroinflammation, cerebral edema, or chronic neuroinflammation (27, 159–161).
Tumor antigen heterogeneity and clonal escape	Multi-targeting strategies such as multi-target CAR-M pools and logic-gated CARs, alongside leveraging the bystander effect (35, 36, 55, 162).	Multi-targeting may compound toxicity risks; logic-gated CAR design is complex; bystander effect efficiency is uncertain in patients (10, 107, 163).
Treatment-related safety risks such as CRS and ICANS	Safety switches such as the iCasp9 system and intracranial local delivery exemplified by convection-enhanced delivery (164–167).	iCasp9 may have a delayed response; local delivery is invasive and may cause local inflammation or increased intracranial pressure (47, 168–170).
Cell manufacturing and *in vivo* delivery bottlenecks	Innovative cell sources including iPSCs for off-the-shelf production and delivery technologies such as MRgFUS for BBB opening and engineering homing receptor overexpression (171–173).	iPSCs carry tumorigenicity and immunogenicity risks; MRgFUS is not yet standardized; overexpressed homing receptors may cause off-target infiltration (174–178).

This table aligns major translational challenges with corresponding engineering or clinical strategies reviewed in the manuscript. The potential limitations of these strategies are also noted.

### 6.1 Functional challenges: phenotypic deactivation and exhaustion

The central challenge lies in the immunosuppressive microenvironment of GBM, which drives phenotypic deactivation and functional exhaustion of CAR-M. This microenvironment is rich in immunosuppressive factors such as IL-4, IL-10, and TGF-β, which activate signaling pathways like STAT3/STAT6 and serve as core drivers polarizing endogenous macrophages toward a pro-tumor phenotype (135, 136). If adoptive CAR-M therapy fails to resist this pressure, functional impairment or phenotypic reversal is likely to occur (137), severely limiting therapeutic efficacy and potentially inducing adverse effects.

The mainstream strategy to address this involves genetically engineering armored CAR-M. For example, preclinical studies suggest that using CRISPR-Cas9 to knock out the negative immune checkpoint gene *SOCS1* can enhance IFN-γ signaling, desensitizing the cells to immunosuppressive signals and promoting stable maintenance of an M1-like state (70). Alternatively, overexpressing *IRF5* or *PPARγ* has been shown to epigenetically reprogram the cells *in vitro*, inherently skewing them toward a pro-inflammatory phenotype (36). More advanced strategies include engineering CAR-M to constitutively express IL-12 or IFN-γ. Evidence from animal models indicates that these factors can act in a paracrine manner to repolarize neighboring TAMs, amplifying the overall anti-tumor immune response (130, 138). Another approach involves expressing CD40L to provide co-stimulatory signals, which has been observed to stabilize the M1 phenotype, repolarize adjacent TAMs, and significantly enhances phagocytic capability while prolonging *in vivo* persistence (19).

However, the translation of these modifications to the clinical setting carries potential risks. Constitutively high expression of inflammatory cytokines raises concerns regarding uncontrollable neuroinflammation, cerebral edema, or cytotoxicity within the intracranial space (139). Moreover, from a long-term perspective, the sustained activation of these engineered CAR-M in the brain might harbor the risk of driving chronic neuroinflammation or autoimmune-like reactions (140, 141), the impact of which on normal neural circuits remains unknown. In summary, without fundamentally mitigating TME-mediated suppression, any subsequent efforts to optimize therapeutic efficacy will face substantial limitations.

### 6.2 Tumor adaptive challenges

Against the backdrop of threat to functional persistence, the challenges of tumor antigen heterogeneity and clonal escape become particularly severe. Even if CAR-M can initially resist TME-mediated suppression, they should contend with substantial subpopulations of tumor cells lacking the target antigen (35). Strategies to overcome this are evolving from single-target approaches toward multi-target synergy and systemic immune activation.

Multi-targeting strategies, such as creating pools of CAR-M or designing logic-gated CARs, offer versatile theoretical solutions (142, 143). Specifically, “OR” logic gates are designed to broaden the recognition spectrum to counter antigen escape, whereas “AND” logic gates aim to enhance specificity by requiring dual-antigen recognition, thereby mitigating the risk of on-target/off-tumor toxicity in the sensitive brain environment (144). While multi-target strategies theoretically expand the cytotoxic scope, they may also compound toxicity risks. Furthermore, validating logic-gated circuits within the intricate *in vivo* environment remains a formidable engineering challenge that has yet to be fully resolved.

However, targeting surface antigens alone may not suffice to eradicate heterogeneous tumors. Therefore, leveraging the potential of CAR-M to stimulate a bystander effect represents a crucial theoretical avenue to address heterogeneity. The successful triggering of this *in situ* vaccination effect in patients faces formidable biological barriers. It relies on a fragile multi-step cascade: CAR-M should successfully infiltrate the tumor and phagocytose a sufficient number of tumor cells; next, they need to survive long enough within the suppressive TME to process and present antigens; and finally, they should effectively activate cytotoxic T cells. Crucially, T cells in GBM patients often exhibit severe functional impairments, exhaustion, or numerical deficiency (145). This creates a critical bottleneck where, even upon successful antigen presentation, the adaptive response may remain inadequate, effectively interrupting the bridging mechanism. Consequently, validating this mechanism and quantifying its actual contribution constitute core objectives for future clinical research.

### 6.3 Clinical applicability challenges

As a potent living cell therapy, particularly when combined with robust armoring strategies, managing safety risks constitutes a fundamental requirement. Potential risks primarily include cytokine release syndrome (CRS) (146), immune effector cell-associated neurotoxicity syndrome (ICANS) (147), and on-target/off-tumor toxicity. To mitigate these risks, incorporating controllable safety switches is crucial. The inducible iCasp9 system has demonstrated the ability to eliminate CAR-M cells upon small-molecule administration in preclinical models, providing a safeguard against severe toxicity (43).

However, iCasp9 may exhibit a delayed response (148). While local delivery techniques, such as convection-enhanced delivery (CED), offer a strategy to drastically reduce the proportion of cells entering the systemic circulation thereby reducing systemic toxicity, they remain invasive and carry procedure-related complications.

Beyond safety, widespread clinical application faces substantial obstacles in large-scale manufacturing and efficient *in vivo* delivery. Regarding manufacturing, primary macrophages possess limited proliferative capacity and exhibit significant donor-to-donor variability, making it difficult to expand them to clinically required doses (149). Regarding delivery, although CAR-M theoretically possess superior homing potential compared to lymphocytes, achieving efficient intracranial infiltration is challenging. Systemic administration faces hurdles beyond the BBB: administered CAR-M may be sequestered by other inflammatory sites, resulting in a significantly lower tumor accumulation than predicted (35). Even if they reach the brain, the integrity of the BBB in GBM patients exhibits individual variability and spatiotemporal heterogeneity (150–152), which can affect the formation and strength of the chemotactic gradient, leading to inconsistent recruitment efficiency of CAR-M.

While engineering strategies, such as overexpression of specific homing receptors (e.g., CXCR2), could theoretically amplify chemotaxis, this approach introduces new risks. It might increase aberrant retention within normal brain structures or promote non-specific infiltration in other organs, potentially causing off-target toxicity. Therefore, addressing these bottlenecks requires improvements at both the cellular source and delivery technology levels. To overcome manufacturing limitations, iPSCs technology offers a platform solution to achieve off-the-shelf production (16, 153). In terms of delivery, alongside the previously mentioned CED technique, systemic administration could employ strategies such as magnetic resonance-guided focused ultrasound (MRgFUS) combined with microbubbles to transiently open the BBB, which has been shown to enhance CAR-M infiltration in animal models (154). However, MRgFUS technology is not yet standardized, and the long-term consequences of BBB opening require further evaluation.

## 7 Future paradigms and perspectives

The future development of CAR-M therapy will not aim to replace existing modalities as a standalone treatment, but rather evolve as a multifunctional platform capable of remodeling the TME, to be strategically integrated into the comprehensive treatment landscape for GBM. This evolution follows a clear logic: target the most pressing clinical challenges, rapidly validate clinical value through combination therapies, and continuously improve efficacy and safety through iterative engineering.

### 7.1 Combination therapy strategies

Combining CAR-M therapy with existing treatment modalities represents the most feasible near-term strategy for rapidly evaluating its clinical value. The core rationale lies in leveraging mechanistic synergy to overcome GBM heterogeneity and immunosuppression. For instance, radiation has been shown to precondition the tumor by disrupting its architecture, releasing tumor antigens, and altering the local chemokine landscape (179). This creates a more favorable environment for the subsequent infiltration and function of infused CAR-M. The CAR-M, in turn, could theoretically clear radiation-damaged tumor cells, potentially enhancing antigen presentation and the “*in situ* vaccination” effect (16, 180). Conversely, it should be noted that radiotherapy can also induce tissue fibrosis and vascular collapse, creating physical barriers that might impede the subsequent infiltration of CAR-M. Therefore, the timing of administration is crucial to balance the benefits of antigen release against the risks of physical exclusion. Furthermore, this combination strategy carries non-negligible risks of overlapping toxicities. Radiotherapy itself can induce acute inflammation and local BBB disruption (181). If superimposed upon the immune activation potentially triggered by CAR-M therapy, the risk of severe local cerebral edema, increased intracranial pressure, and even acute neurological deficits may be significantly heightened (182, 183). Therefore, meticulous management of the treatment sequence is paramount. Administering CAR-M after the acute phase of radiotherapy, once the initial inflammatory peak has subsided, is proposed as a potential approach to mitigate these risks (184, 185). Furthermore, this strategy should be accompanied by close neurological assessment and radiographic monitoring throughout the treatment course to balance synergistic efficacy against neurotoxicity.

Parallel to radiotherapy, combination with immune checkpoint inhibitors (ICIs) aims to achieve sequential activation of innate and adaptive immunity. Here, CAR-M acts as the innate immune engine, responsible for reversing local immunosuppression and recruiting T cells, while PD-1/PD-L1 inhibitors are designed to remove the functional constraints on these recruited T cells (186). A central uncertainty of this strategy is the potential for significantly amplified immune-related adverse events (irAEs), particularly neurotoxicity. It is hypothesized that the inflammatory *milieu* generated by CAR-M activation may further disrupt BBB integrity, potentially allowing more circulating antibody-based drugs to enter the central nervous system (187, 188). This could precipitate or exacerbate serious conditions such as ICANS. To mitigate this risk, adopting a sequenced administration protocol rather than simultaneous dosing may be a safer approach (189). This involves administering CAR-M first and then introducing ICIs only after preliminary confirmation *via* biopsy or imaging that the CAR-M has successfully remodeled the TME and recruited endogenous T cells. By temporally separating the two phases of immune activation, this approach theoretically aims to reduce the risk of early, explosive severe neurotoxicity.

### 7.2 Next-generation engineering strategies

However, the foundation of combination therapy rests upon the controllability and robustness of the CAR-M platform itself. Existing constitutively active CAR-M still faces the risks of functional deactivation or excessive activation within the complex human environment. Consequently, the long-term developmental direction involves leveraging synthetic biology tools to create next-generation, intelligent CAR-M capable of sensing and responding to the TME. Notably, the vast majority of these designs remain conceptual or in early *in vitro* validation stages. Their feasibility, safety, and necessity for clinical translation are all unresolved core issues. For instance, using hypoxia-inducible promoters to regulate CAR expression aims to enhance tumor localization specificity (190). However, the *in vivo* precision of such systems faces severe challenges: physiological hypoxic zones exist natively in the brain, and hypoxia within tumor regions is notoriously non-uniform (191), potentially leading to off-target CAR expression or insufficient intensity. Similarly, developing CAR systems that can be externally regulated by small-molecule drugs faces significant clinical translation bottlenecks (33). These include the long-term stability of synthetic genetic circuits in the human body, the potential immunogenicity triggered by repeated drug administration (192), and the pharmacokinetic challenge of whether small-molecule drugs can reach effective concentrations intracranially. Even more complex designs involve engineering multifunctional CAR-M that actively recruit and activate T cells, for example, by enabling them to secrete T cell chemokines or cytokines (55, 193). Such strategies harbor unpredictable risks within the enclosed intracranial space. Uncontrolled chemokine expression might disrupt neuro-immune homeostasis, potentially triggering uncontrollable excessive inflammation or a “cytokine storm,” the consequences of which for normal neural circuitry remain largely unknown (194).

### 7.3 Clinical translation paradigms

Given the high level of uncertainty inherent in both combination therapies and intelligent technologies, a prudent clinical translation pathway prioritizing mechanistic validation is paramount. During this data-scarce phase, early-phase clinical trials should be designed not merely to confirm safety, but as powerful biological exploration platforms dedicated to answering a fundamental question: Does CAR-M operate in patients as predicted? Consequently, we advocate for implementing a risk-controlled strategy in first-in-human trials, mandating a pre-specified, multidimensional analysis plan for mechanistic biomarkers. This plan should systematically evaluate three core aspects: first, directly verifying the intracranial homing efficiency and intratumoral persistence of CAR-M *via* scheduled tumor biopsies or *in vivo* cell tracking imaging (195); second, precisely analyzing the phenotypic status of CAR-M within the authentic TME through flow cytometry and single-cell RNA sequencing—specifically, assessing whether they maintain the “locked” M1-like polarization or undergo functional deactivation; finally, monitoring dynamic changes in the intratumoral and peripheral immune cell repertoire to identify objective evidence of elicited adaptive immune responses.

Collectively, the future paradigm for CAR-M therapy encompasses a spiral development process evolving from combination applications to intelligent upgrades, and ultimately to mechanism-driven iterative translation. Through this phased, validation-focused strategy, we can potentially transform this cutting-edge platform from a promising concept into tangible clinical benefits for GBM patients.

## 8 Conclusion

In summary, the core value of CAR-M therapy lies in its role as a multifunctional immunotherapy platform capable of coordinately addressing the delivery, immunosuppressive, and heterogeneity challenges in GBM, rather than merely serving as a cytotoxic weapon. This review elucidates that its successful clinical translation is fundamentally a dynamic systems biology problem: whether the engineered cells can achieve stable, durable integration and functional output within the complex tumor ecology of patients. Confronting severe challenges such as TME-induced phenotypic deactivation, antigen escape, and *in vivo* delivery bottlenecks, future developments should abandon linear thinking focused on singular technological optimizations. Instead, the field should embrace a new translational paradigm centered on mechanistic validation and iterative advancement. By deepening our understanding of macrophage biology and the TME, and by profoundly integrating intelligent engineering, combination strategies, and pragmatic clinical research frameworks, we can potentially evolve CAR-M from a promising concept into a critical component within the comprehensive therapeutic arsenal for GBM.
